# A Physics-Consistent Framework for Semiconductor Device Reliability Including Multiple Degradation Mechanisms

**DOI:** 10.3390/mi17030320

**Published:** 2026-03-04

**Authors:** Joseph B. Bernstein, Tsuriel Avraham, Bin Wang

**Affiliations:** 1 Department of Electrical and Electronic Engineering, Ariel University, Ariel 40700, Israel; josephbe@ariel.ac.il (J.B.B.); tsuriel2@gmail.com (T.A.); 2 The Department of Electrical Engineering, Nanjing University of Posts and Telecommunications (NUPT), Nanjing 210023, China

**Keywords:** semiconductor reliability, physics-of-failure (PoF), multi-time-of-life (MTOL), BTI, TDDB, HCI, additive hazard modeling, lifetime extrapolation, SiC, GaN, reliability standards, power electronics

## Abstract

Reliability assessment of semiconductor devices increasingly requires the consideration of multiple degradation mechanisms acting simultaneously over long stress durations. Conventional lifetime qualification and prediction approaches rely on simplified assumptions that can obscure the interpretation of measured degradation data and lead to large uncertainty when extrapolated over many orders of magnitude in time. A consistent analytical framework is therefore required to relate measured degradation behavior to meaningful reliability metrics. This work presents a general framework for semiconductor device reliability that is consistent with established reliability theory and explicitly accommodates multiple competing degradation mechanisms, consistent with modern JEDEC reliability standards. The framework presented here separates physical degradation processes from analytical representations used to interpret experimental data, allowing the effect of independent mechanisms to be combined without imposing an implied physical model. Degradation behaviors exhibiting sublinear time dependence, which are commonly observed across device technologies, are discussed within this context. We show that common data interpretation practices can introduce systematic errors when ssublinearkinetics are present, particularly regarding lifetime extrapolation. A reformulated analytical representation is introduced that improves clarity and robustness in lifetime extraction while remaining fully compatible with standard reliability theory. This framework supports more consistent reliability assessment and more credible lifetime prediction across materials, devices, and operating conditions.

## 1. Introduction

Semiconductor reliability modeling has long relied on simplified empirical techniques that may not fully capture the physics of degradation in advanced technologies [1]. For decades, standards such as MIL-HDBK-217F [2] and Siemens SN29500 have employed multiplicative π-factor methodologies, in which environmental, temperature, and quality factors are combined to estimate an overall acceleration factor (AF) [3,4,5]. While convenient, this framework is strictly valid only when a single dominant failure mechanism governs device behavior and the associated acceleration factor is well characterized.

These assumptions can be violated in modern wide-bandgap devices and in advanced-node silicon technologies employing high-κ gate dielectrics, where multiple degradation paths may coexist and compete [6,7,8,9,10,11,12]. Recent surveys of CMOS reliability trends have likewise highlighted growing uncertainty in prediction accuracy as device architectures and materials continue to evolve [13]. In such regimes, applying single-mechanism models without explicit uncertainty treatment can introduce systematic error.

Zero-failure qualification tests (e.g., JEDEC HTOL) can provide useful screening information; however, interpreting them as precise field failure-rate predictors requires care. The Poisson upper limit yields only a bound on the observed failure rate, while the mapping from stress to use conditions depends strongly on correct mechanism identification and acceleration-factor uncertainty [14,15]. JEDEC guidance (e.g., JEP122G) explicitly recognizes that multiple mechanisms may be simultaneously active and that their contributions should be treated accordingly [15].

Legacy single-mechanism extrapolations therefore do not capture the parallel contributions of multiple degradation mechanisms—such as bias temperature instability (BTI), time-dependent dielectric breakdown (TDDB), hot-carrier injection (HCI), and electromigration (EM)—each of which exhibits distinct field and temperature sensitivities.

Physics-of-Failure (PoF) modeling combined with Multiple Temperature Operational Life (MTOL) testing provides an alternative framework. Instead of assigning multiplicative correction factors, each degradation mechanism is described by its own kinetic process and corresponding hazard function. The total system hazard then becomes the sum of the individual contributions,

(1) $$\lambda_{total}(t)=\sum_{i}\lambda_{i}(t)$$

yielding an additive model consistent with both reliability theory and device physics. This formulation is explicitly aligned with JEP122G, which states that when multiple failure mechanisms and corresponding acceleration factors are present, a proper summation technique (e.g., sum of failure rates) should be used [15].

The purpose of this paper is to identify the limitations of existing approaches and to propose a unified, data-driven alternative anchored in measured degradation kinetics. The goal is to demonstrate a framework consistent with JEDEC guidance for properly combining competing failure mechanisms.

## 2. Limitations of Current Standards

### 2.1. Interpreting JEDEC FIT Estimates Outside Their Validity Ranges

In the JEDEC zero-failure methodology, devices are stressed for a fixed time $t_{stress}$ with N samples and zero observed failures. The failure rate is then bound by the Poisson upper limit:

(2) $$FIT=\frac{-\ln\left(1-CL\right)}{N\;t_{stress}AF}\times 10^{9}$$

where CL is the confidence level (commonly 60% or 90%) [14,15]. This procedure can create an impression of certainty—suggesting a finite FIT rate even with no measured failures—while masking substantial uncertainty in the acceleration factor (AF) and thus, extrapolated FIT. For mechanisms that follow power-law time dependence (*t^n^*), extrapolation from a few hundred hours to 10 years can amplify even small uncertainty in the assumed power law, *n*.

Furthermore, JEDEC’s implicit assumption of a single governing mechanism may likely be inconsistent with its own guidance in JEP122G, which explicitly requires a proper sum-of-failure-rates (additive hazard) treatment. In practice, this inherent gap within the same standard documents can remain unresolved when qualification data are interpreted using a single effective acceleration factor. The methodology described in what follows outlines a pathway to make reliability predictions more transparent by combining mechanism isolation with explicit uncertainty treatment.

### 2.2. The π-Factor Multiplicative Model

The MIL-HDBK-217 and Siemens SN29500 reliability standards both adopt a multiplicative model:

(3) $$\lambda_{system}=\lambda_{b}\times \pi_{T}\times \pi_{E}\times \pi_{Q}\times \ldots$$

where each π-term represents a correction for temperature, environment, or quality, etc. This formulation implies statistical independence and proportional scaling of all factors; an assumption that is often not physically justified when multiple degradation processes coexist. As device technologies evolved from planar silicon to high-κ/metal-gate CMOS and now to wide-bandgap and Gate All-Around (GAA) materials, degradation mechanisms have become increasingly nonlinear and simultaneously active. The π-factor model does not readily represent phenomena such as trap-generation kinetics, electric-field-driven breakdown, or charge trapping with recovery. Furthermore, the π- factor is not even listed for these newer technologies and processes.

### 2.3. False Confidence from Zero Failures

A further issue is the optimistic interpretation of survivor bias induced by guaranteed “zero-failure” results. In practice, published qualification reports nearly universally report zero failures per published accelerated HTOL test. Engineers may interpret the absence of observed failures as evidence of robustness, when it may instead reflect insufficient stress duration or sample size, obscuring the actual potential expected lifetime.

A generalization of the reliability function, for any non-constant λ(*t*) (hazard function that changes with time), is expressed in a form suitable for linearization and parameter extraction [3,4,5]. This formulation allows us to define each failure mechanism as a single average λ*_i_* per mechanism, *i*, allowing a linear combination of multiple mechanisms where each mechanism is identified by its effective failure rate, resulting in the MTOL mechanism combination (matrix) approach [16,17,18]. We thus assume that the reliability per mechanism uses the standard reliability theory expression relating survival probability to the integrated hazard rate for each mechanism,

(4) $$R_{i}(t)=e^{-\int_{0}^{t}\lambda_{i}(\tau )\;d\tau }$$

which is the standard reliability theory expression relating survival probability to the integrated hazard function [16]. When $\lambda_{i}$ and its stress-to-use mapping are poorly constrained, long-term extrapolation becomes correspondingly uncertain.

This uncertainty is particularly important when the rate itself is determined because of “zero failure” survivor bias. This JEDEC standard-based approach [14,15] is particularly relevant for large-scale infrastructure deployments, where modest modeling and extrapolation errors can have enormous material, technical, and economic consequences, as recognized in the JEDEC documents. This follows because when multiple mechanisms exist, one cannot combine acceleration factors for multiple mechanisms into a single multiplicative acceleration factor, such as commonly seen with the π factor approach of (Equation (3)). This is clear since each mechanism is affected so differently by each stress component and these effects are highly nonlinear, even exponential or according to a power law.

### 2.4. Relation to Existing BTI Reliability Models and Novelty of the Present Work

Bias Temperature Instability (BTI) degradation has traditionally been described using an empirical power-law dependence of the form:

(5) $$\Delta V_{th}\left(t\right)=A\;t^{n},$$

where the pre-factor A captures bias and temperature dependence and the exponent, n, reflects the underlying defect kinetics. This functional form has been widely reported in the literature for Si, SiC, and GaN technologies [6,7,8,9,10,11,12].

The present work does not introduce a new physical degradation law. Instead, it looks at the conventional BTI power-law as a representative expression that can be combined in a linearized representation with respect to time that eliminates the need to assume an initial threshold voltage $V_{th}(t=0)$, which is experimentally inaccessible due to ultra-fast trapping effects.

The contribution of this work is the development of a linearization methodology that enables robust extraction of the time exponent n directly from measured data without baseline ambiguity. This approach provides a unified analytical framework applicable across different semiconductor technologies, while allowing for distinct physical degradation mechanisms and parameters.

## 3. The Physics-of-Failure (PoF) Framework

The PoF methodology replaces empirical factors with measurable physical parameters, including activation energy $E_{a}$, voltage/current acceleration parameter γ, and time exponent n [16]. Each mechanism is modeled individually with its characteristic kinetics. Representative forms include:

• BTI: $\Delta V_{th}=A\;t^{n}\;V^{\gamma }e^{-E_{a}/kT}$;
• TDDB: $t_{f}=A_{TDDB}\;e^{E_{a}/kT}\;e^{\gamma V}$;
• HCI: $\lambda_{HCI}=\lambda_{0}\;I^{\gamma }e^{-E_{HCI}/kT}$.

The total cumulative hazard (failure) function becomes

(6) $$F(t)=1-R(t)=1-exp\left(-\int_{0}^{t}(\lambda_{BTI}(\tau )+\lambda_{TDDB}(\tau )+\lambda_{HCI}(\tau ))\;d\tau \right)$$

where $\lambda_{BTI}(t)$, $\lambda_{TDDB}(t)$, and $\lambda_{HCI}\left(t\right)$ denote the mechanism-specific time-dependent hazard rates associated with bias temperature instability (BTI), time-dependent dielectric breakdown (TDDB), and hot-carrier injection (HCI), respectively. This formulation enables direct estimation of reliability metrics such as mean time to failure (MTTF) under specified use conditions, reflecting the real-time competition between mechanisms. Accordingly, the total failure rate is obtained by summing the individual mechanism contributions under the relevant stress conditions (Equation (6)). A single zero-failure test, even when repeated across stresses, generally cannot disentangle competing mechanism rates without additional mechanism-isolating information.

### 3.1. Advantages over Empirical Standards

Empirical standards that employ π-factor multiplication often start with a “base” failure rate derived from limited qualification data (usually zero failure, HTOL-based) and then modify it with empirical π factors [16]. When multiple mechanisms contribute, compounding factors can lead to overly optimistic predictions because the approach effectively treats diverse stress effects as if they accelerate a single mechanism, rather than summing distinct rate processes. The result of separating mechanisms and combining them additively has the following advantages over many current standard approaches:

• Transparency: Each parameter has a measurable physical meaning.
• Scalability: Independent parameters can be updated as technology evolves.
• Predictive Validity: Additive hazards modeling aligns with observed Weibull mixtures in experimental failure data.

When a zero-failure test and its resulting upper-bound in uncertainty is combined with π-factor multiplication across disparate mechanisms, the resulting estimate has no statistical meaning and lacks a mechanism-based justification, since the multiplicative factors correspond to their effects on different mechanisms. For example, temperature and voltage can accelerate BTI while not affecting HCI. Also, high switching frequency leading to high currents will accelerate HCI while not affecting BTI.

Thus, we see that different failure processes should not be multiplied together as if they act on a single underlying mechanism. Furthermore, there is no specific mechanism reported in these standards so they cannot be verified in the first place. Not only that, but the initial “base” failure rate is reported as if it reflects an implicit single-mechanism assumption since that is the only justification for multiplying factors in the first place [16]. Thus, at the very least, a multiple-temperature operational life test (MTOL) is required to separate and calibrate mechanism-specific contributions [17,18].

### 3.2. Applicability of the Linearization Framework to Si, SiC, and GaN Technologies

Although the proposed linearization framework is applied to Si, SiC, and GaN devices, this does not imply identical physical degradation mechanisms across these technologies. In silicon MOSFETs, BTI is primarily governed by interface state generation and oxide charge trapping. In SiC MOSFETs, oxide defects and near-interface traps dominate, often exhibiting stronger temperature and field dependence. In GaN-based devices, degradation is frequently associated with buffer trapping, surface states, and field-induced charge redistribution. Regardless of the specific physical origin of the degradation, the extrapolated time to fail can be determined through the PoF-based model for that phenomenon in the specific material system.

The linearization method introduced in this work is agnostic to the microscopic origin of degradation. It provides a mathematical framework for extracting sublinear kinetics from experimental data, while allowing each technology to retain its own dominant physical mechanisms and parameter values. As such, the framework unifies data analysis without enforcing a common physical model. Most importantly, the tendency for degradation follows a sublinear power law, where the exponent, n<1, where values are reported as low as 1/6 to 1/8 for BTI and even for HCI as well as for SiC and GaN power device degradation [6,7,8,9,10,11,12], as will be developed next.

## 4. Time Extrapolation and the Power-Law Model

The time dependence of many degradation processes, particularly Bias Temperature Instability (BTI), is often approximated by a sublinear power-law relationship between threshold voltage shift, *V_th_*, and stress time, *t*, to the power of *n*, as expressed in (Equation (5)).

### 4.1. The Power-Law Relation

Here, A is a stress-dependent pre-factor, and n is the empirical time exponent, typically ranging from 0.1 to 0.4. This model captures the observed sublinear degradation under constant stress conditions [19,20].

However, the exponent n is not physically universal. It arises from the convolution of multiple underlying physical processes (i.e., charge trapping/detrapping, hydrogen diffusion, and trap relaxation) and therefore varies with device structure, stress conditions, and measurement protocols [21,22]. For extrapolation of TTF based on accelerated life tests, the assumption must be that n is independent of voltage and temperature. However, this exponent has been reported to depend on voltage and temperature stress. Thus, substantial modeling errors may be introduced when extrapolating to operating lifetimes to calculate TTF. This is only one of the sources of error in extrapolating time to fail from accelerated test data.

### 4.2. Extrapolation Sensitivity

For a given failure threshold $\Delta V_{th,crit}$, the time-to-failure (TTF) can be derived by inverting the power-law expression:

(7) $$TTF={\left(\frac{\Delta V_{th,crit}}{A}\right)}^{1/n}$$

This equation highlights the exponential sensitivity of lifetime prediction to the value of n. Even small changes in the time exponent can yield orders-of-magnitude variation in the projected lifetime when extrapolating over order of magnitude in time. Such sensitivity becomes especially problematic in accelerated lifetime testing, where devices are stressed at elevated conditions (e.g., 1000 h at 175 °C) and extrapolated to use conditions (e.g., 10 years at 100 °C). In these cases, the effective acceleration factor becomes highly nonlinear and uncertain [1].

**Figure 1:**
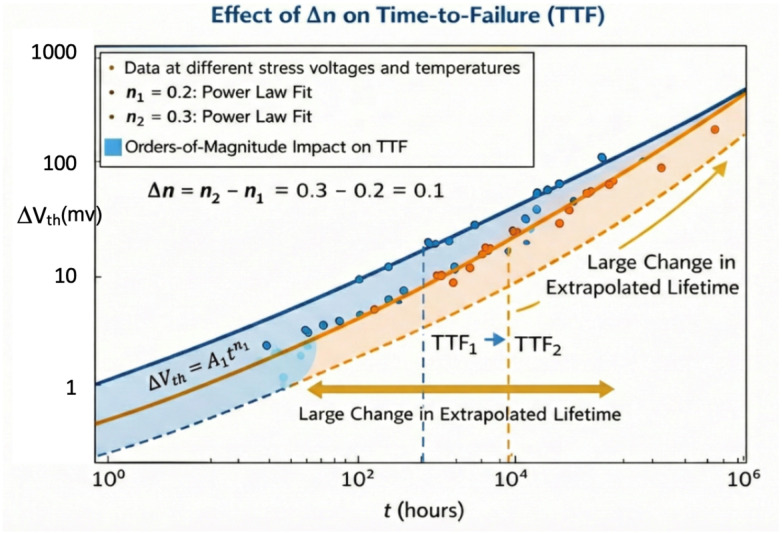
Sensitivity of lifetime extrapolation to variations in time exponent n. Even small changes in slope yield large errors in projected time-to-failure (TTF).

This effect is visualized in Figure 1, where small deviations in slope (i.e., Δn=0.1) between extrapolated fits result in vastly different TTF predictions—despite originating from the same data set.

### 4.3. Temperature and Field Dependence

Both the pre-factor A and exponent n are effectively functions of gate voltage ($V_{G}$) and temperature (T). The pre-factor A typically follows an Arrhenius-type relation modulated by electric field stress. A representative form is:

(8) $$A=A_{0}\cdot e^{-E_{a}/kT}\cdot e^{\gamma V_{G}}$$

where $E_{a}$ is the activation energy, γ is the field acceleration coefficient, and $V_{G}$ represents the gate-induced field across the oxide. Importantly, the exponent n itself has been observed to decrease with increasing temperature and field, due to mechanisms such as enhanced recovery, trap saturation, and field-assisted detrapping [21,22,23]. This implies that the commonly cited “universal” value of n is simply not a material constant, but rather a byproduct of specific stress protocols. This has very important implications for lifetime extrapolation [1].

## 5. The BTI Plotting Dilemma and Our Correction

### 5.1. The False-Origin Problem

Traditional BTI lifetime characterization plots the threshold-voltage shift ΔV_th_ versus stress time and fits a straight line on log-log axes, with its slope corresponding to the time exponent *n*. In practice, defining the initial reference voltage V_*th*0_ can be challenging because applying stress perturbs ${∆V}_{th}(t)=V_{th}(t){-V}_{th0}$ on very short time scales (microseconds or faster), and conventional measurement systems may not clearly capture this earliest transient. As a result, extracted *n* values can be highly sensitive to the assumed initial condition and to the measurement protocol (including recovery), leading to scatter across published studies. Therefore, literature-reported *n* values should be interpreted with caution unless the experimental protocol and the handling of early-time transients are clearly documented and validated for the technology under study.

### 5.2. Bernstein’s Modified Plotting Method

Bernstein [1] proposed a formulation that addresses the plotting error due to the sensitivity of power-law plots to the selection of an initial value (at *t* = 0). Rather than plotting a difference in threshold voltage (as is normally done for BTI data extrapolation), Bernstein [1] proposed reparametrizing the time (X) axis. Rather than relying on an unmeasurable initial threshold voltage (because the initial transient is too fast) at zero time, the method fits the absolute threshold voltage Vth as a function of $t^{1/m}$ including a second-order term in $t^{2/m}$. The exponent *m* is chosen such that the coefficient of the second-order term, in this Taylor-like expansion, approaches zero. This will yield a near-as-possible linear relation in the transformed X-axis giving a proper linearly fitting parameter *A* that is not dependent on any assumed *V_th_*_0_:

(9) $$V_{th}\left(t\right)=V_{th0}+A\;\left(t^{1/m}\right)+B\left(t^{2/m}\right)$$

In this formulation, the y-axis plots the absolute *V_th_*(*t*) rather than Δ*V_th_*, which removes any sensitivity to the assumed initial value *V_th_*_0_. A least-squares fit identifies m such that the curvature term is minimized (B ≈ 0), improving parameter identifiability and reducing false-origin bias, leading to a realistic and consistent power-law time exponent, $n=\frac{1}{m}$. This plotting technique assures that the power, *n*, is properly determined by most of the data and not weighted improperly by noisy initial values [1].

This correction reduces bias from unmeasured early transients and enables more stable extraction of *V_th_*_0_, *A*, and *m*, improving long-term extrapolation of time to fail (TTF). A similar transformation can be applied to other parameters that exhibit power-law time evolution. By solving for B = 0, we know that the extrapolation is linear over the timeframe and will lead to a realistic extrapolation of TTF for the given accelerated life test.

### 5.3. Physical Interpretation

In the corrected plotting framework, the power-law exponent $n=\frac{1}{m}$ emerges from the fit transformation, where m is selected to linearize the degradation trajectory over time. This representation implicitly bypasses the unmeasurable early transient regime—typically dominated by near-instantaneous charge trapping near the oxide interface—which occurs within microseconds of stress application and is generally invisible to conventional test systems.

By redefining the time axis in this way, the proposed method effectively mitigates false-origin bias and improves long-term extrapolation stability. However, it is important to clarify that the exponent n derived from this procedure is a mathematical parameter, not a direct probe of a specific physical process such as near-interface trap filling.

This formulation enables extrapolated kinetics that are self-consistent with both major BTI physical frameworks:

• In defect-centric models, it emerges from the statistical superposition of capture/emission events at oxide and interface traps with widely distributed time constants [24,25].
• In the reaction-diffusion (R-D) model, power-law behavior arises from hydrogen transport and interfacial reactions under diffusion-limited conditions [22,26,27].

Thus, the modified plotting method is mechanism-agnostic: it provides a robust empirical extrapolation approach while remaining compatible with both diffusion-limited and reaction-limited interpretations. Furthermore, this form of power-law degradation has been observed in a broad range of semiconductor materials and can be viewed as an actual empirical trend, rather than a signature of any single underlying mechanism. As such, the corrected model facilitates extrapolation back to an effective initial time (t=0) while reducing sensitivity to measurement protocol variability and early-time artifacts.

## 6. Critique of the π-Factor Model

The π-factor model originated in the 1960s military and industrial reliability handbooks (e.g., MIL-HDBK-217) to estimate equipment failure rates using multiplicative empirical modifiers. Each factor (temperature, environment, quality, stress) was represented as a series of multipliers as corrections to some baseline failure rate $\lambda_{b}$ (Equation (3)). While effective for macroscopic components (capacitors, relays, etc.), this model is difficult to apply directly to semiconductor degradation physics, where distinct microscopic mechanisms can coexist and interact. This use of a single composite acceleration factor implicitly assumes a single effective mechanism driven by a combination of stressors [16]. When multiple mechanisms compete under a given set of operating conditions, their failure rates are better treated as distinct processes and combined statistically (e.g., via additive hazards).

### 6.1. A Common Pitfall in Multiplicative Aggregation

When one multiplies π-factors, he implicitly treats diverse stress effects as if they accelerate a single underlying failure mechanism in the same manner. In semiconductors, however, each mechanism (BTI, TDDB, HCI, electromigration, etc.) follows a distinct kinetic law with its own stress sensitivity [28]. Consequently, purely multiplicative aggregation does not accurately represent the combined hazard when multiple mechanisms are active. In some regimes, it can produce estimates ranging from overly optimistic to overly conservative, depending on the underlying assumptions and calibration. More importantly, the resulting value lacks a clear reliability theory interpretation and mechanism-based justification expected of a physics-based model.

The correct formulation for coexisting mechanisms is additive:

(10) $$\lambda_{total}=\sum_{i}\lambda_{i}=\lambda_{BTI}+\lambda_{TDDB}+\lambda_{HCI}+\ldots$$

This additive law follows directly from reliability theory, in which the total system hazard equals the sum of independent hazard functions.

### 6.2. Quantitative Example

Consider a MOSFET with BTI and TDDB characteristic lifetimes of 10^5^ and 10^8^ h, respectively. A multiplicative π-factor approach that assumes a single effective mechanism can mask the presence of a second mechanism and may yield an apparent lifetime that is inconsistent with an additive-hazard interpretation. In contrast, under an additive model (as recognized in JEP122G when multiple mechanisms are active), the effective lifetime is obtained by summing the corresponding failure rates,

(11) $$\frac{1}{TTF_{\mathrm{eff}}}=\frac{1}{10^{5}}+\frac{1}{10^{8}}\Rightarrow TTF_{\mathrm{eff}}\;\sim 10^{5}\;h$$

which can differ by orders of magnitude relative to a single-mechanism extrapolation, depending on the relative rates. This simple example illustrates the risk of applying system-level empirical aggregation to semiconductor-level multi-mechanism reliability without mechanism separation and independent validation.

Interestingly, despite clear guidance in JEP122G [15], which requires a proper sum-of-failure-rates treatment when multiple mechanisms are active, multiplicative aggregation is still used ubiquitously in modern industrial practice. As device scaling accelerates and wide-bandgap materials introduce new degradation paths, legacy approaches can yield highly optimistic FIT estimates when applied outside their validated ranges.

## 7. Multi-Mechanism Reliability Interaction

Contrary to the assertion of a multiplicative π factor assumption, modern power and mixed-signal devices rarely degrade through a single mechanism [15,27,28,29,30]. In SiC MOSFETs, Bias Temperature Instability (BTI), Time-Dependent Dielectric Breakdown (TDDB), and Hot-Carrier Injection (HCI) all evolve simultaneously and interact nonlinearly [6,7,8,9,10,11,12]. Thus, total hazard rate must be expressed as the sum of the individual contributions to the total failure rate:

(12) $$\lambda_{\mathrm{total}}(T,V,F)=\lambda_{\mathrm{BTI}}(T,V,F)+\lambda_{\mathrm{TDDB}}(T,V,F)+\lambda_{\mathrm{HCI}}(T,V,F)+\ldots \lambda_{i}(T,V,F)$$

**Figure 2:**
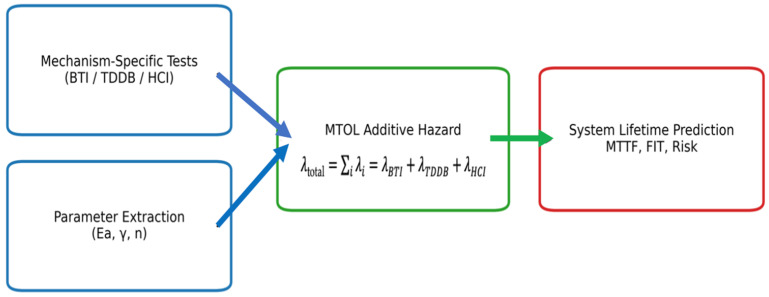
Mechanism-isolated MTOL reliability workflow. Stress-mode-specific accelerated testing (BTI, TDDB, HCI) enables extraction of physics-based parameters, including activation energy $E_{a}$, field-acceleration factor γ, and time exponent n. The resulting mechanism-specific hazard rates are then combined using the additive hazard formulation to obtain the total system failure rate and corresponding lifetime metrics (MTTF, FIT, and risk).

Each *λ_i_*(*t*) depends on stress history, recovery dynamics, and process activation energies. The lambdas are determined by (4) so that each mechanism is characterized by its unique average extrapolated failure rate a function of operational conditions, i.e., Temperature, Voltage or Frequency (T, V, F, …). Because these mechanisms are temperature- and voltage- dependent in different ways, their relative dominance can change over time and stress, producing crossover effects that can undermine a single-mechanism extrapolations. The cumulative failure distribution then becomes a mixture of Weibull distributed populations:

(13) $$F\left(t\right)=1-\exp\left[-\sum_{i}{\left(\frac{t}{\eta_{i}}\right)}^{\beta_{i}}\right]$$

where $\eta_{i}$ and $\beta_{i}$ are the scale and shape parameters for mechanism *i*. Accurate prediction therefore requires individual calibration of each component. The MTOL system is described in further detail elsewhere [29,30]. This MTOL summation method is built into a practical workflow as illustrated in Figure 2 [3,4,5].

## 8. Implications for AI Datacenter Hardware

The shift toward AI-accelerated computing has exposed limitations in legacy reliability modeling frameworks. Modern datacenter hardware—including large GPU arrays, SiC/GaN-based power converters, and high-frequency switching elements—operates at elevated thermal densities and under near-continuous electrical stress. Under these conditions, even small modeling inaccuracies can propagate into significant system-level reliability risk [28,31].

Local thermal coupling, exacerbated by high utilization and limited heat-dissipation paths, can lead to self-heating that accelerates thermally activated degradation mechanisms such as BTI, TDDB, and electromigration. Compounding this effect, dynamic workloads introduce non-stationary stress conditions—including rapid bias cycling and fluctuating current densities—rendering traditional JEDEC-style constant-stress acceleration assumptions increasingly inaccurate [16,17,18,19,20,21,22].

The growing deployment of highly scaled CMOS technologies, such as GAA nanosheets, in AI and datacenter workloads further amplifies the importance of accurate multi-mechanism reliability prediction. In large-scale datacenters comprising thousands of similar chips, even small modeling errors can propagate into large-scale system reliability projections [31].

In tightly optimized systems, even modest performance degradation—such as slight threshold shifts or timing violations—can result in operational faults or even complete computational failure. Worse, built-in array-level redundancy may mask early-stage degradation, leading to undetected risk accumulation until cascading failure occurs at a larger scale. Under these conditions, lifetime prediction must reflect the independent contributions of coexisting mechanisms. An additive hazard formulation becomes the most physically consistent means of summing failure rates, according to the real JEDEC requirement:

(14) $$\frac{1}{{MTTF}_{\mathrm{system}}}=\sum_{j}\frac{1}{{MTTF}_{j}}=\sum_{j}\int_{0}^{\infty }\lambda_{j}(t)f_{j}(t)\;dt$$

In contrast, the traditional multiplicative model conflates disparate stress effects into a single synthetic acceleration factor, often assuming independence where it does not exist.

(15) $${MTTF}_{\pi }=\frac{1}{\lambda_{b}\prod_{i}\pi_{i}}$$

This can result in non-conservative FIT estimates that are inconsistent with statistical field returns and fail to capture the nuanced degradation behavior of advanced systems.

**Figure 3:**
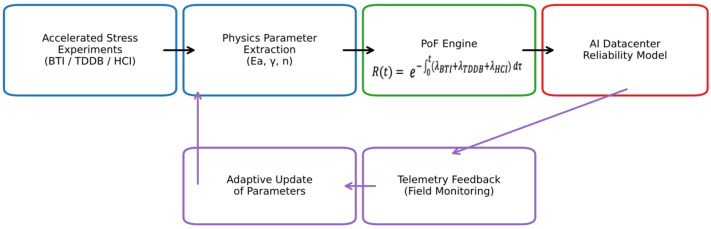
Closed-loop physics-of-failure (PoF) reliability framework for advanced semiconductor and AI datacenter hardware. Accelerated stress experiments and parameter extraction feed the PoF engine, where mechanism-specific hazard rates are combined to compute system reliability. Field telemetry enables adaptive parameter updating, supporting dynamic, application-aware lifetime prediction under realistic operating conditions.

Figure 3 gives a schematic representation of an integrated physics-of-failure (PoF) framework for semiconductor reliability modeling. The left panel illustrates laboratory-based accelerated testing under distinct stress conditions (BTI, TDDB, HCI), each governed by its own physical kinetics. These are independently modeled to extract degradation parameters (e.g., $E_{a}$, γ, n), which are then summed using additive hazard logic to produce a composite failure rate $\lambda_{\mathrm{total}}(t)$. The right panel depicts system-level application in AI datacenter hardware, where PoF modeling informs lifetime prediction and operational risk assessment across complex workloads. This methodology contrasts with traditional π-factor-based approaches by isolating mechanisms and quantifying their cumulative impact on failure distribution (see Figure 3).

## 9. Practical Implementation and Experimental Validation

### 9.1. Implementation of MTOL

To implement a predictive and transparent reliability framework for next-generation semiconductor systems, the following modeling workflow is proposed:

• **Mechanism Isolation**
  Conduct stress-mode-specific accelerated testing for BTI, TDDB, and HCI to independently extract kinetic parameters, such as activation energy $E_{a}$, field acceleration factor γ, and time exponent n.
• **Uncertainty Quantification**
  Report confidence intervals, rather than nominal values, for each extracted parameter. This enables downstream propagation of modeling uncertainty into system-level predictions.
• **Additive Hazard Reconstruction**
  Use the MTOL (Multiple Temperature Operational Life) framework to combine stress-mode-specific hazard rates into a total system hazard:
  $$\lambda_{\mathrm{total}}=\lambda_{\mathrm{BTI}}+\lambda_{\mathrm{TDDB}}+\lambda_{\mathrm{HCI}}+\ldots$$
• **Model Validation**
  Please refer to Table 1. Compare the model’s predicted failure distribution against both accelerated test results and field-level use data, closing the loop between physics-based modeling and real-world behavior.

**Table 1:** Summarizes the contrast between conventional JEDEC-style modeling and the proposed physics-of-failure paradigm.

Feature	Traditional (JEDEC/MIL)	Proposed (PoF)
Mechanism Focus	Single dominant mechanism	Multiple concurrent mechanisms
Mathematical Logic	Multiplicative π-factors	Additive hazard rates (λ_total_ = ∑ λ_i_)
Extrapolation Basis	Empirical zero-failure limits	Physically derived kinetic laws
Accuracy	Assumption-dependent	Mechanism-aware and parameterized

The corrected plotting framework proposed in [1] supports this paradigm by removing false-origin artifacts in BTI extrapolation. Applying it to wide-bandgap devices enables more reliable extraction of $n(V_{G},T)$, thereby avoiding artificial overestimation of robustness. Looking ahead, we recommend integrating machine-learning surrogates trained on physically meaningful parameters. Such models can enable near-real-time reliability prediction during AI hardware operation, bridging physics, statistics, and live telemetry to support adaptive, risk-aware system management. When mechanisms exhibit coupling (e.g., shared defect populations or stress-history dependence), the framework can be extended using state-dependent hazard rates or interaction terms while retaining the additive competing-risk structure.

### 9.2. Demonstration from Measured FinFET MTOL Data

We apply the proposed framework to measured degradation data from 16 nm FinFET FPGA devices obtained using previously published Multi-Temperature Operational Life (MTOL) testing [17]. MTOL measurements provide in situ monitoring of ring-oscillator frequency $F_{RO}$ during stress, enabling extraction of time kinetics from fully functional hardware under realistic operating conditions. This example contrasts two interpretations of the same dataset: a conventional quarter-power assumption and a data-driven extraction of the power-law exponent.

**Figure 4:**
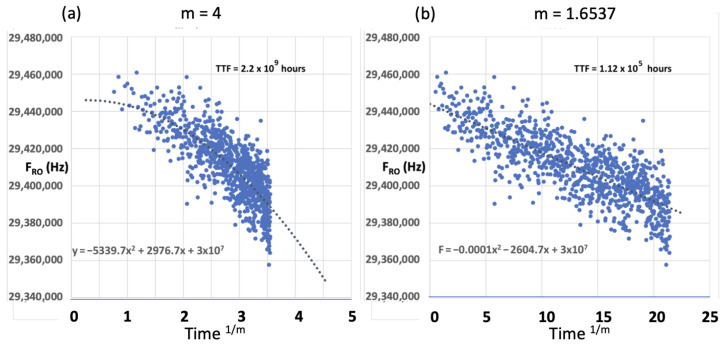
Demonstration of exponent sensitivity and curvature control using measured 16 nm FinFET MTOL degradation data plotted as ring-oscillator frequency $F_{RO}$ (Hz) versus transformed time. (**a**) Conventional representation assuming m=4 (n=0.25) yields an extrapolated $TTF\approx 2.2\times 10^{9}\;h$. (**b**) Curvature-reduced fit (quadratic term suppressed) yields m≈1.65 and $TTF\approx 1.12\times 10^{5}\;h$.

Figure 4a shows a conventional representation in which the time exponent is assumed to follow quarter-power behavior, corresponding to $n=\frac{1}{m}=0.25\;$(i.e., m=4). Under this assumption, extrapolation yields an apparent time-to-failure of $TTF\approx 2.2\times 10^{9}\;h$. However, this very long projected lifetime arises from curvature implicit in the conventional fit and from sensitivity to the assumed exponent. In contrast, applying the linearization methodology described here yields a more physically consistent estimate of $TTF\approx 1.12\times 10^{5}\;h$ (≈11 years). This result highlights the importance of using an appropriate axis transformation that linearizes the data for reliable extrapolation.

This example highlights the central claim of this work: in regimes exhibiting sublinear kinetics and curvature, lifetime projection may be influenced not only by device physics but also by the analysis representation and the implicit assumptions embedded in the fitting procedure. The two projections differ by approximately four orders of magnitude despite being derived from the same measured dataset.

The proposed linearization and curvature-control approaches provide a practical means to stabilize extraction of the effective time exponent and thereby reduce extrapolation uncertainty without requiring a priori commitment to a single universal n-law across all operating conditions and technologies. While the present dataset is BTI-dominated, the same methodology is applicable to any degradation observable exhibiting sublinear time dependence, including HCI- and TDDB-driven parameter shifts.

## 10. Conclusions

This paper argues that reliability modeling for next-generation semiconductor devices and AI-scale hardware increasingly requires a unified framework that remains valid under multi-mechanism, multi-stress operation. Traditional qualification standards and handbook-style models provide valuable screening and comparability, but their direct use as lifetime predictors can be problematic when mechanism dominance shifts, acceleration factors are uncertain, and multiple mechanisms degrade the devices concurrently. A physics-of-failure (PoF) paradigm—built on mechanism-isolated testing, explicit uncertainty quantification, and additive hazard reconstruction—offers a principled path toward more predictive reliability assessment for nano-scale Silicon and SiC/GaN power technologies all being utilized in today’s AI-scale systems.

**Key takeaways are:**

- Multi-mechanism reliability is naturally represented by additive hazards, where mechanism hazards are summed to obtain the total hazard.
- Zero-failure accelerated test outcomes should be interpreted with uncertainty, particularly when acceleration factors, stress-to-use mapping, or mechanism coverage are not uniquely established.
- Empirical multiplicative scaling rules should be used cautiously and, when applied, should be calibrated and validated against mechanism-specific degradation and hazard data.
- Power-law extrapolation benefits from careful treatment of early-time transients; corrected plotting strategies reduce false-origin bias and improve identifiability of degradation kinetics.
- A practical PoF workflow—mechanism isolation, parameter extraction with confidence bounds, hazard reconstruction, and validation—enables more transparent and auditable lifetime prediction.
- For AI-scale hardware, coupling PoF models with telemetry and data-driven updating can support operational reliability management, including risk-aware derating and maintenance planning.

Future work should address correlated mechanisms, stress-history effects under realistic workloads, and standardized reporting of uncertainty so that reliability predictions remain comparable while being physically grounded.

## Data Availability

The original contributions presented in this study are included in the article. Further inquiries can be directed to the corresponding author.
